# Cryptococcal meningitis in a patient with VEXAS syndrome: a case report

**DOI:** 10.1186/s41927-026-00648-6

**Published:** 2026-05-09

**Authors:** Michelle Chun-Ping Lin, Thomas Day, Stefan Lammerink, Syed B. Ali

**Affiliations:** 1 Department of Clinical Immunology and Allergy, Flinders Medical Centre, Flinders Drive, Bedford Park, South Australia Australia; 2 School of Medicine and Public Health, Flinders University, Bedford Park, South Australia Australia; 3 Department of Haematology, Flinders Medical Centre, Bedford Park, South Australia Australia; 4 Department of Microbiology and Infectious Diseases, Flinders Medical Centre, Bedford Park, South Australia Australia; 5 Infectious Diseases Department, Women’s and Children’s Hospital, North Adelaide, South Australia Australia; 6 Faculty of Medicine, Adelaide University, Adelaide, South Australia Australia

**Keywords:** VEXAS, Infection, Cryptococcus, Invasive fungal infection, Immune dysregulation, Antimicrobial prophylaxis

## Abstract

**Background:**

Vacuoles, E1 enzyme, X-linked, autoinflammatory somatic (VEXAS) syndrome is a recently described multisystem disease. Opportunistic infections in VEXAS syndrome are increasingly being recognized and attributed to multiple factors, including immune dysregulation due to an aberrant ubiquitination pathway, secondary effects from chronic immunosuppressive treatments and underlying patient comorbidities. Given the limited consensus on antimicrobial prophylaxis, clinical practice is heterogeneous, with varying treatment outcomes.

**Case presentation:**

Herein, we report a rare case of cryptococcal meningitis in a patient with treatment-refractory VEXAS syndrome, on chronic moderate-dose corticosteroids (an average of 20 mg prednisolone equivalent per day) and ruxolitinib. The patient initially presented with acute confusion and fevers, with elevated inflammatory markers and worsening cytopenia. The differential diagnoses included an acute flare of VEXAS syndrome and intercurrent infection. Further testing revealed the presence of *Cryptococcus neoformans* in the cerebrospinal fluid. Treatment with prolonged antifungal therapy was successful.

**Conclusion:**

The case raises important considerations, firstly regarding antimicrobial - particularly antifungal - prophylaxis given limited evidence in the literature, secondly a prompt diagnosis and treatment of cryptococcal infections, and finally regular interval screening for opportunistic infections in this vulnerable patient cohort. A multidisciplinary approach to care is required to improve morbidity and mortality from atypical infections in VEXAS syndrome.

## Background

Vacuoles, E1 enzyme, X-linked, autoinflammatory somatic (VEXAS) syndrome is a recently described multisystem disease arising from a somatic mutation in the *UBA1* gene [1]. The estimated prevalence is rare, occurring in 1 in 13,591 individuals across all age groups [2]. The *UBA1* gene encodes for a vital protein in the ubiquitination pathway, and mutation leads to impairment in protein degradation and cellular signalling, resulting in profound cellular stress and inflammation [3]. As ubiquitination is essential in many cell lines, clinical presentation of VEXAS syndrome is heterogeneous and involves multiple organ systems. Bone marrow involvement is common, presenting with multilineage cytopenia and progression to concurrent myelodysplastic syndrome (MDS) in around one-third of patients, further contributing to morbidity and mortality [4].

The persistent proinflammatory state requires immunosuppressive treatment. In the literature to date, this disease is often refractory to treatment, resulting in prolonged and intensive use of immunosuppressive agents. Patients with VEXAS syndrome are generally older and hence have accrued comorbidities. The addition of necessary immunosuppressive therapy and bone marrow insufficiency, in the form of cytopenia or MDS, significantly increases their risk of opportunistic infections. Furthermore, it is being increasingly recognised that immune dysregulation in VEXAS syndrome may also play a role [5]. For example, invasive fungal infections (IFI) and legionella infections have been reported, independent of immunosuppressive treatments [6].

In the absence of clear guidelines on antimicrobial prophylaxis, along with the treatment-refractory nature of the disease, clinicians must be vigilant for infection in patients with VEXAS syndrome. A range of opportunistic viral, bacterial, and fungal infections have been reported, with a recent systematic review highlighting the high prevalence and heterogeneity in this cohort [7].

Herein, we report a rare case of cryptococcal meningitis in a patient with treatment-refractory VEXAS syndrome without concurrent MDS. The case provides important considerations in early recognition, diagnosis and management of patients with this complex multi-system disease.

## Case presentation

A 69-year-old male was managed in the immunology clinic for polyarteritis nodosa (PAN) with per oral (p.o.) mycophenolate 1 g twice daily (BD) and prednisolone 15 mg daily. His other background history included an 8-year history of ankylosing spondylitis, with a recent switch to subcutaneous (s.c.) etanercept after limited improvement on s.c. secukinumab.

A year after commencing treatment for PAN, he developed breathlessness and cough over several months. Etanercept was withheld, however symptoms progressed, leading to hospitalisation. On admission, he had macrocytic anemia and was in type 1 respiratory failure. Imaging demonstrated extensive ground glass opacities (GGO) without evidence of a concurrent infective or vascular process. The breathlessness and GGO responded to p.o. prednisolone 15 mg with taper over a fortnight; however, macrocytic anemia persisted with hemoglobin (Hb) of 72 g/L (135–175 g/L) and mean corpuscular volume (MCV) of 102 fL (80–98 fL). Investigations including hematinic and haemolytic screens were unremarkable. A bone marrow biopsy was reported as normal.

Over the following 12 months, systemic symptoms with PAN persisted which included lower limb paraesthesia and weakness. A positron emission tomography (PET) scan suggested features of vasculitis in the popliteal arteries. Intravenous (i.v.) tocilizumab treatment was initiated thereafter, but persistent symptoms led to treatment cessation after four months. Prednisolone dosing remained at 15 mg, and trimethoprim/sulfamethoxazole 160 mg/800 mg (TMP-SMX) half tablet daily was initiated for *Pneumocystis jirovecii* prophylaxis.

Within weeks of ceasing tocilizumab, he was diagnosed with extensive bilateral deep vein thrombosis (DVT) on Doppler ultrasonography, and apixaban was initiated. A progress scan at 6 months showed persistent extensive thrombi, with lifelong anticoagulation advised by the haematologist. On a routine outpatient follow-up, bloods demonstrated a trilineage cytopenia with Hb 125 g/L, white cell count (WCC) of 3.13 × 10^9^/L (4.00–11.00 × 10^9^/L) and platelet count (PLT) of 99 × 10^9^/L (150–450 × 10^9^/L), which did not resolve on withholding mycophenolate. Comprehensive assessment for secondary causes of cytopenia was unremarkable, including negative human immunodeficiency virus (HIV) serology and no radiological evidence of malignancy on CT and PET imaging.

**Fig. 1:**
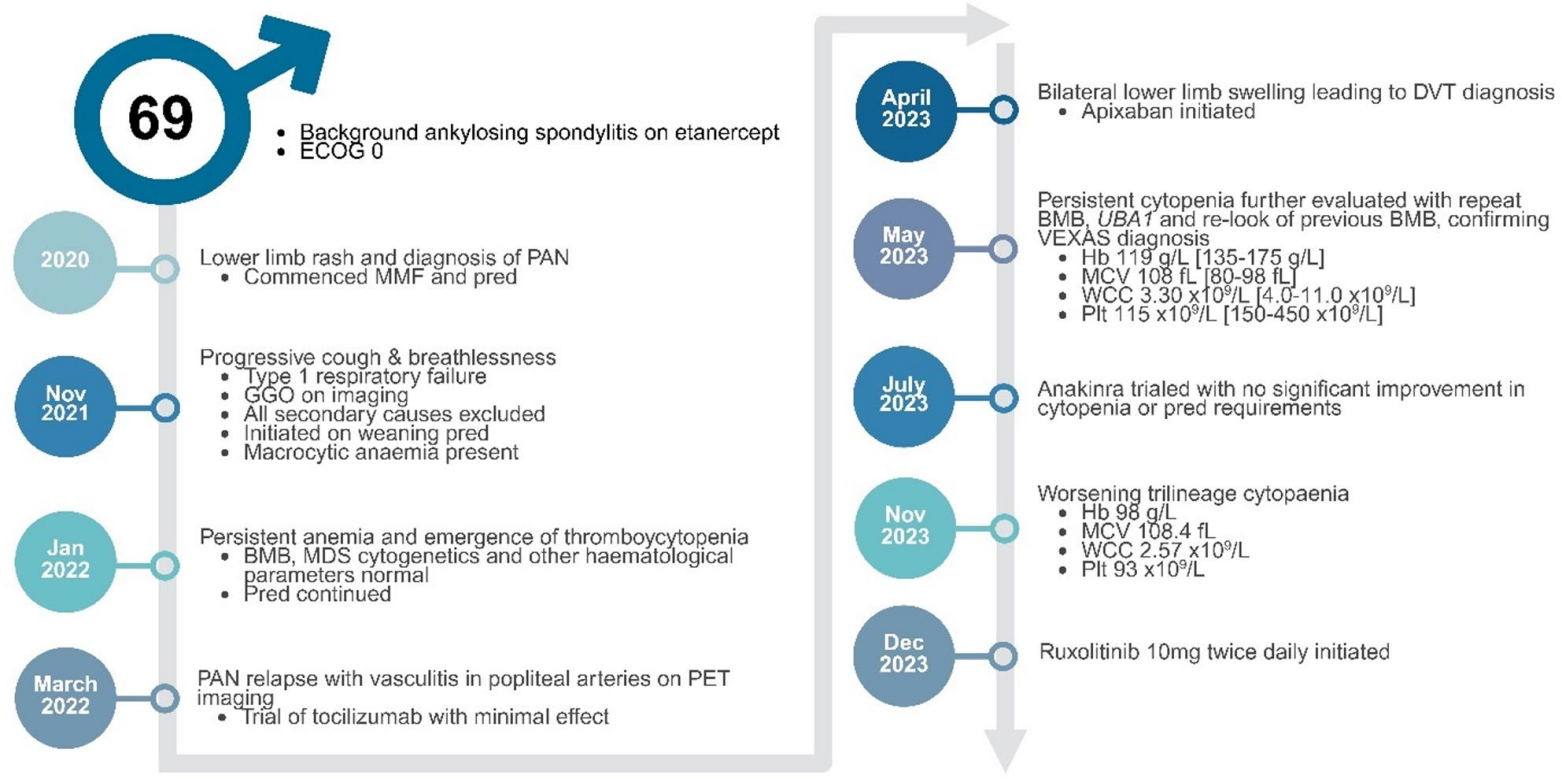
Timeline of events leading to VEXAS syndrome diagnosis. Abbreviations: BMB, bone marrow biopsy; DVT, deep vein thrombosis; Eastern Cooperative Oncology Group, ECOG; GGO, ground glass opacities; Hb, hemoglobin; MCV, mean corpuscular volume; MDS, myelodysplastic syndrome; MMF, mycophenolate mofetil; PET, positron emission tomography; PLT, platelet count; pred, prednisolone; WCC, white cell count

**Fig. 2:**
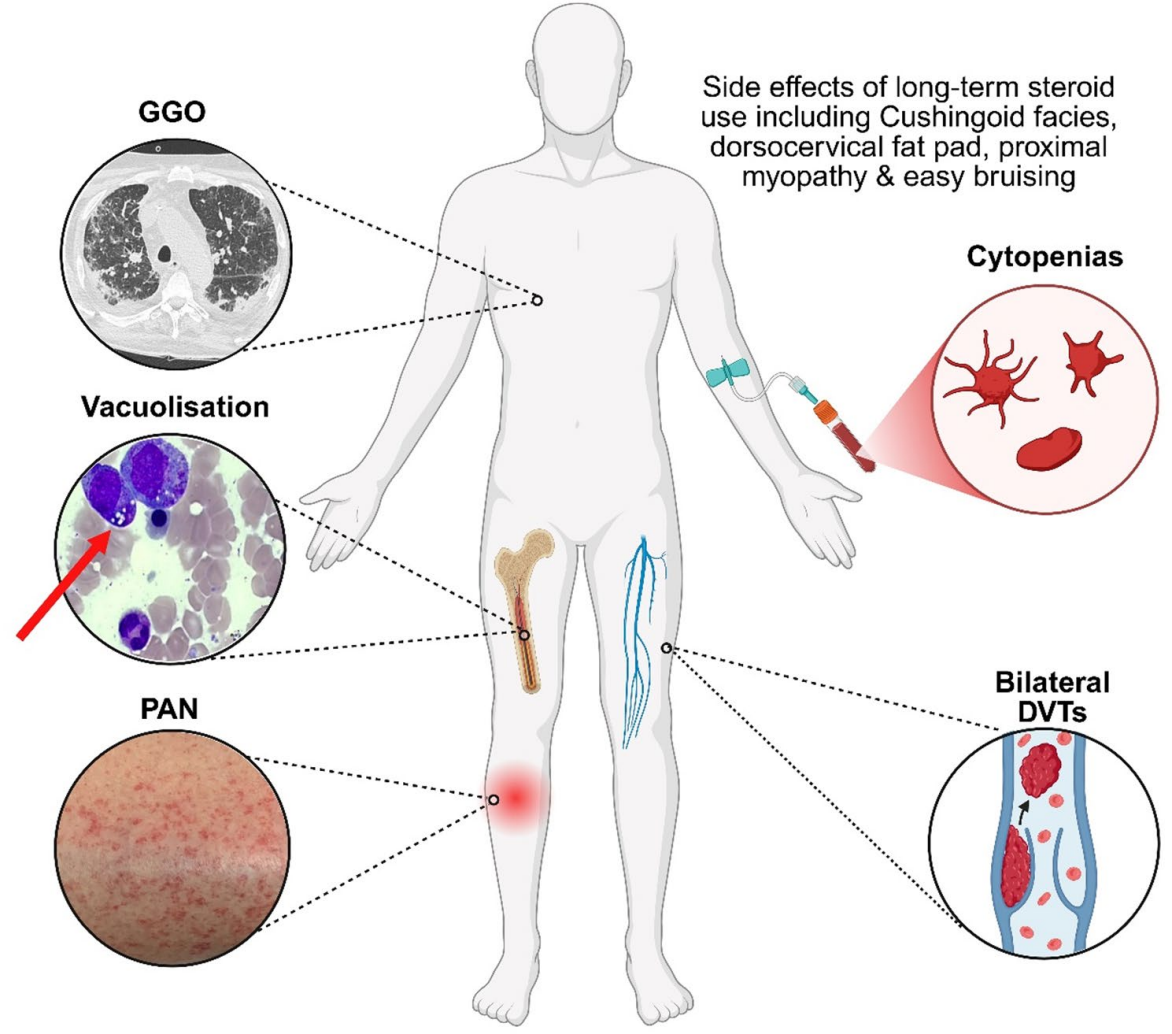
Spectrum of clinical features predating diagnosis of VEXAS syndrome. Red arrow pointing to the cytoplasmic vacuoles in myeloid cells on bone marrow biopsy for this patient (Wright-Giemsa stain, 100x oil). Abbreviations: GGO, ground glass opacity; PAN: polyarteritis nodosa; DVTs, deep vein thrombosis

The combination of persistent cytopenia with macrocytosis, PAN and DVT in an older male led to the consideration of VEXAS syndrome given its recent description (Fig. 1). Peripheral blood testing confirmed a somatic mutation in the UBA1 gene; c.121 A > G p.Met41Val. Bone marrow biopsy was repeated and confirmed characteristic cytoplasmic vacuolation (Fig. 2). The haematology department re-reviewed the bone marrow biopsy performed 18 months earlier and confirmed vacuolation. Following prolonged and protracted symptoms over years, a unifying diagnosis of VEXAS syndrome was reached (Fig. 1).

Prednisolone treatment was increased to 20 mg daily, and after a six-month trial of anakinra, compassionate access to p.o. ruxolitinib at 10 mg BD was approved. He continued TMP-SMX and was commenced on p.o. valaciclovir 500 mg daily for viral prophylaxis. His immunizations were up to date. His functional status at this time was equivalent to an Eastern Cooperative Oncology Group (ECOG) score of 1. His CD4 count was 270 cells/mm^3^ (500–1500 cells/mm^3^) and his C-reactive protein (CRP) was 3 mg/L (0.0–8.0 mg/L).

Over the next four months, cytopenia persisted and was refractory to prednisolone dosage below 17.5 mg daily. Importantly, CRP remained normal. He developed complications of long-term corticosteroid therapy including Cushingoid facies, increased interscapular fat pad, proximal myopathy and osteopenia (Fig. 2). His HbA1c was 6.4% (≤ 7%).

Around 12 months later, he developed subacute onset of fevers, malaise, headache and vomiting over a period of around two weeks. On presentation, he was febrile (39 °C) with Glasgow coma scale of 13 (E3, V4, M6), hypoxic (SaO_2_ 95% on 2 L oxygen) with unilateral lower limb swelling. Investigations revealed worsening cytopenia: Hb 80 g/L, WCC 2.05 × 10^9^/L (including neutrophil count 1.10 × 10^9^/L [1.80–7.50 × 10^9^/L]) and PLT 67 × 10^9^/L. Inflammatory markers were elevated: CRP 253.4 mg/L and erythrocyte sedimentation rate (ESR) > 120 mm/hr (1–15 mm/hr). Imaging for venous thromboembolism demonstrated stable appearance of chronic thrombi in his lower limbs. Magnetic resonance imaging (MRI) brain with gadolinium contrast identified subtle multifocal punctate enhancing foci in both cerebellar hemispheres, suggestive of early changes of rhombencephalitis. As a lumbar puncture could not be performed immediately due to anticoagulation and thrombocytopenia, multidisciplinary input involving immunology, haematology and infectious diseases was sought. The consensus opinion was to concurrently treat a possible VEXAS flare with pulse methylprednisolone, while also providing empiric infective meningoencephalitis cover with i.v. benzylpenicillin, i.v. ceftriaxone and i.v. acyclovir.

A serum cryptococcal antigen (CrAg) was sent, but results only became available (positive titre 1:16) just prior to the lumbar puncture, which was performed on day five of admission. Notable findings of the lumbar puncture included an opening pressure of 27 cmH_2_O and an inflammatory cerebrospinal fluid (CSF) assessment: protein 0.89 g/L (0.15–0.45 g/L), glucose 3.8 mmol/L (2.2–5.5 mmol/L), 3 × 10^6^ polymorphonuclear cells/L (0 × 10^6^/L), 44 × 10^6^ mononuclear cells/L (0–5 × 10^6^/L) and 10 × 10^6^ erythrocytes/L (0 × 10^6^/L). Organisms resembling cryptococci were seen on India Ink staining, with CSF CrAg strongly positive at a titre of 1:512. The CSF subsequently cultured *Cryptococcus neoformans (C. neoformans)* after 3 days.

Extensive testing for other infective pathogens was negative. CSF cultures on specific media for bacterial, mycobacterial and other fungal pathogens were negative. CSF polymerase chain reaction (PCR) testing for enteroviruses, varicella zoster virus, herpes simplex virus, Japanese encephalitis virus, Murray Valley encephalitis virus, *Streptococcus pneumoniae*, *Neisseria meningitidis*, and *Listeria monocytogenes* was negative. Blood PCR testing for cytomegalovirus and human herpesvirus 6 was negative. Serological testing for Toxoplasma, syphilis, and HIV was negative. Serological testing for Japanese encephalitis virus indicated evidence of previous infection, with no evidence of acute infection (positive serum IgG, negative serum and CSF IgM).

**Fig. 3:**
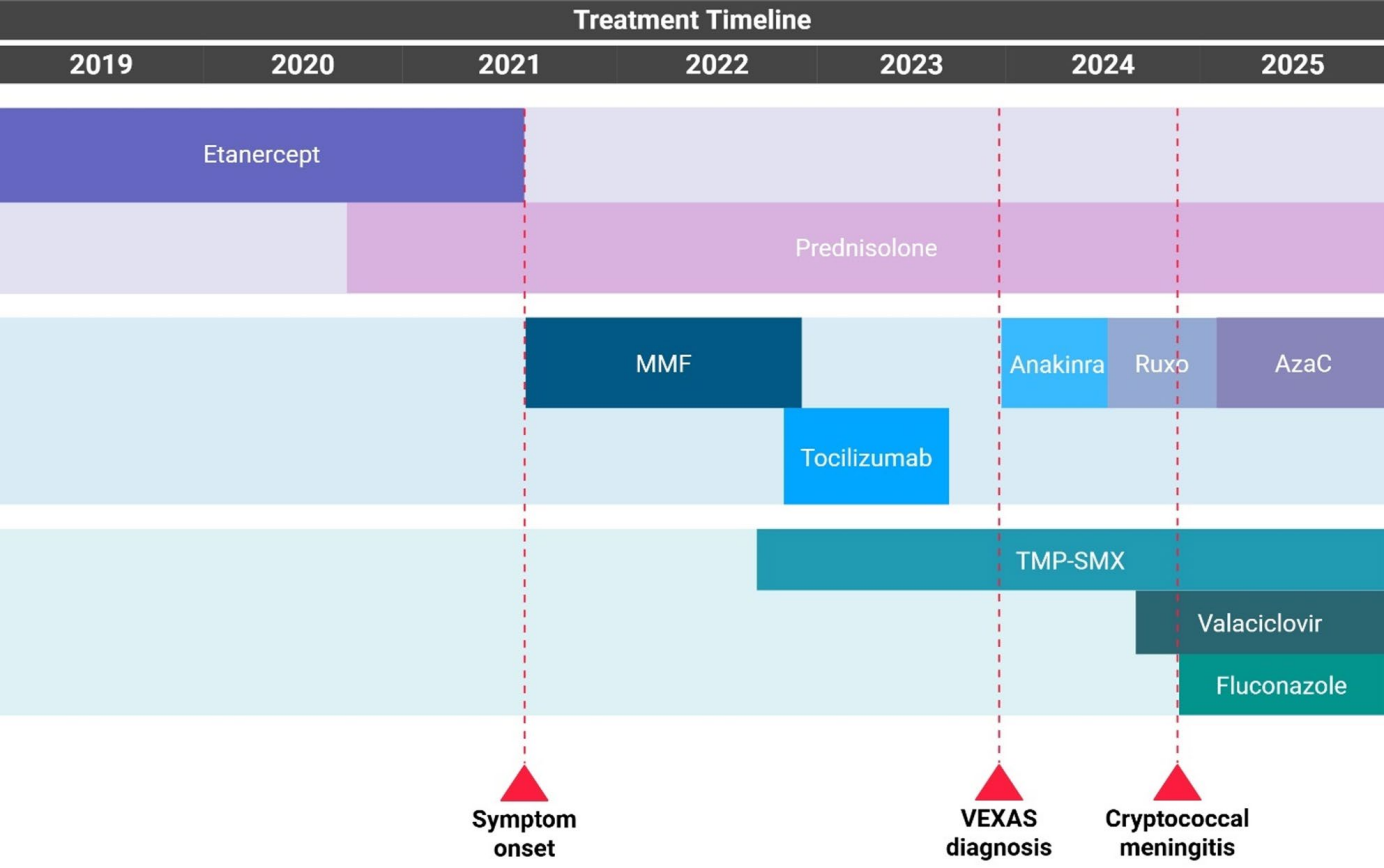
Treatment timeline in relation to symptom onset, VEXAS syndrome and cryptococcal meningitisdiagnosis. Abbreviations: AzaC, azacitidine; MMF, mycophenolate mofetil; Ruxo, Ruxolitinib; TMP-SMX, trimethoprim, sulfamethoxazole

A diagnosis of cryptococcal meningitis was made and induction therapy commenced (Fig. 3) with i.v. liposomal amphotericin B (L-AMB) 300 mg daily (3 mg/kg) and p.o. flucytosine 2 g 6-hourly, in line with global guidelines [8]. Other antimicrobials were discontinued.

After 10 days of treatment, L-AMB was discontinued and i.v. fluconazole 400 mg daily initiated. This change was initially made due to concerns for L-AMB toxicity with rising creatinine from 80 to 193 µmol/L (60–110 µmol/L), and was further supported after antimicrobial susceptibility results became available. Antifungal susceptibility testing was performed in accordance with the Clinical and Laboratory Standards Institute (CLSI) methodology [9]. The minimum inhibitory concentrations (MICs) were 2 mg/ml for amphotericin B, 8 mg/ml for fluconazole, and 1 mg/ml for flucytosine, respectively. CLSI does not explicitly report true susceptibility breakpoints for *C. neoformans* but does publish epidemiological cut-off values (ECVs). Based on these ECVs, the amphotericin MIC was considered non-wild type, indicating the likely presence of an acquired resistance mechanism, while the fluconazole and flucytosine MICs were considered wild-type, indicating the likely absence of an acquired resistance mechanism [9].

Flucytosine was adjusted for renal function and serum levels at a regimen of 2 g BD. Therapeutic drug monitoring for flucytosine was routinely performed, and levels were in the range of 25–120 mg/L throughout the dose interval, which is considered optimal for treatment. Ruxolitinib dose was reduced to 5 mg BD due to an interaction with fluconazole, while prednisolone dose was reduced to 50 mg with weaning following course of pulse methylprednisolone. TMP-SMX and valaciclovir were continued.

A repeat lumbar puncture on day 14 of cryptococcal therapy demonstrated a persistently elevated opening pressure of 27.5 cmH_2_O and a raised CSF protein of 0.67; cultures were negative. This included the absence of cryptococci on India Ink staining and no growth on fungal or bacterial culture (Table 1). Fluconazole was increased to 800 mg daily due to an improvement in renal function, and dual antifungal therapy was continued for a month. This was followed by p.o. fluconazole monotherapy at 800 mg daily for a further 8 weeks, then 200 mg daily indefinitely as maintenance therapy and secondary prophylaxis. Ruxolitinib therapy was ceased given lack of overall efficacy and prednisolone monotherapy was continued (Fig. 3).

For the management of elevated intracranial pressure, a multidisciplinary approach led to the decision to pursue active surveillance rather than routine therapeutic lumbar puncture or CSF shunt placement. This decision was made after weighing up risks versus benefits of repeated intervention in a patient with challenging thrombosis – recurrent venous thromboembolism warranting anticoagulation – but also persistent thrombocytopenia (reduced to less than 50 × 10^9^/L during admission). In addition, given only modestly elevated intracranial pressure and no papilledema on fundoscopy, the benefit of CSF drainage was thought to be limited. Acetazolamide was not used given its negative implications in patients with cryptococcal meningitis [10].

**Table 1:** Summary of results from CSF examinations

Results of CSF examination	Day 4 of admission	Day 18 of admission
Appearance	Clear	Clear
Protein level (0.15–0.45 g/L)	0.89 g/L	0.67 g/L
Glucose level (2.2–5.5 mmol/L)	3.8 mmol/L	4.4 mmol/L
Polymorphonuclear cells (range: 0 cells)	3 × 10^6^/L	0
Mononuclear cells (range: 0–5 cells)	44 × 10^6^/L	31 × 10^6^/L
Red blood cells (range: 0 cells)	10 × 10^6^/L	2 × 10^6^/L
Culture	No growth on aerobic and anaerobic incubation after 4 days	No growth on aerobic and anaerobic incubation after 4 days
Fungal culture	*Cryptococcus neoformans*	No fungal elements detected on direct microscopy No fungal growth after 4 weeks
AFB	Negative	Not performed
CrAg Titre	1:512	1:32

AFB: acid-fast bacilli. CrAg: cryptococcal antigen

While the headaches and fevers resolved, his functional recovery was hampered by profound fatigue, which was multifactorial in the context of prolonged hospitalisation, transfusion-dependent anemia, and steroid-induced myopathy. Despite rehabilitation, he experienced a significant decline in function and was discharged to a residential care facility with ECOG status of 3. Given poor functional status, a haematology consensus was reached for consideration of low-dose azacitidine over haematopoetic stem cell transplantation.

## Discussion

This report describes a rare case of cryptococcal meningitis in a patient with VEXAS syndrome without concurrent MDS. Treatment with numerous lines of immunosuppressive therapies, including prolonged exposure to corticosteroids, as well as the immune dysregulation due to impaired ubiquitination, collectively predispose patients with VEXAS syndrome to serious opportunistic infections [10]. Such infections, particularly disseminated to the central nervous system, have a pronounced impact on patient function and overall quality of life [5, 11]. Therefore, clinicians need to be vigilant in screening, diagnosing and consolidating guidelines for infection prophylaxis in this vulnerable patient cohort to improve treatment outcomes. While there are no specific guidelines pertaining to antifungal prophylaxis in patients with VEXAS syndrome, the key learning points from this case are as follows: [1] there is an increased risk of opportunistic infections, [2] prompt investigations and treatments are required, and [3] antifungal prophylaxis should be considered in these patients to prevent irreversible sequelae.

Cryptococcus is one of the most common non-viral causes of meningitis in the immunocompromised host, with considerable mortality associated given its insidious presentation that often results in delays in diagnosis and treatment [11]. To our knowledge, this is the first case of *C. neoformans* meningitis in a patient with VEXAS syndrome. On review of the literature to date, only one case of *C. neoformans* pneumonia has been identified from France [6, 7]. One additional case of cryptococcal pneumonia (species not reported) has also been described, although the infection predated the diagnosis of VEXAS syndrome by several months [12]. Historically, patients with acquired immunodeficiency syndrome (AIDS) from HIV were most susceptible, however the rise in immunomodulatory therapies have led to an epidemiologic shift in affected patient cohorts [11, 13]. In non-HIV cohorts, risk factors include extended course of high-dose corticosteroids (although the exact dose and duration remain unclear) and exposure to several immunosuppressive therapies [14, 15]. These include calcineurin inhibitors (e.g. tacrolimus) and mammalian target of rapamycin inhibitors (e.g. sirolimus) in solid-organ transplant recipients and rare cases described with anti-tumour necrosis factor alpha, sphingosine-1-phosphate receptor, anti-CD52 and anti-Bruton’s tyrosine kinase inhibitors [11, 14, 16]. There are also specific patient cohorts with increased risk, including those with inborn errors of immunity and sarcoidosis [11, 16, 17]. Interestingly, the rates of Cryptococcus infections were low among stem-cell transplant recipients, a finding likely attributable to the routine use of azole antifungals as prophylaxis in these patients [17–19].

Multiple factors contribute to the risk of infection in VEXAS syndrome, including intrinsic immune dysregulation attributable to the disease itself and extrinsic effects of immunosuppressive therapy [5, 20]. This was described in a cohort of 813 patients with VEXAS, noting that opportunistic infections occurred even in patients not on immunosuppressive therapy [21]. On a cellular level, in vitro studies have demonstrated evidence of monocyte exhaustion with diminished counts and aberrant chemokine receptor expression, alongside upregulation of proinflammatory cytokines involved in inflammasome activation such as IL-1β and IL-18 [5, 20]. It is thus imperative that infection be considered in the management of patients with VEXAS syndrome, irrespective of immunosuppression.

Our patient was refractory to a range of biologic disease-modifying antirheumatic drugs (DMARDs), frequently requiring steroid uptitration to prevent worsening cytopenia. On review, his cumulative steroid burden equated to an annual prednisolone dose of 1300 mg (≥ 0.3 mg/kg/day). The immunosuppressive effects of corticosteroids are well documented, which affect both the innate and adaptive immune system. Some of these effects include impaired neutrophil phagocytic activity and toll-like receptor signalling, as well as reducing macrophage, monocyte and natural killer cytotoxicity through downregulation of cytokine production [14, 15]. In addition, treatment with ruxolitinib, a JAK1/2 inhibitor of the JAK/STAT pathway, further predisposes to viral reactivation by interfering with natural killer cell maturation and by blocking dendritic cell differentiation and antigen presentation. A systematic review identified herpes zoster infection (1336 patients affected per year, odds ratio 5.20, 95% CI, 1.27 -21.18) as the most common ruxolitinib-associated infectious complication in MDS patients, for which antiviral prophylaxis can be considered [22]. Moreover, the risk of hepatitis B virus and latent tuberculosis reactivation with JAK inhibitors (JAKi) has also been raised, and pre-treatment screening is recommended for at-risk groups [23]. There are no guidelines on antifungal prophylaxis for patients on JAKi. Cases of ruxolitinib-associated IFI are rare in comparison to herpes zoster infections with only twelve reported cases of cryptococcosis in MDS patients receiving ruxolitinib, one of which had a diagnosis of chronic myelomonocytic leukaemia on combination azacitidine and cytarabine [16]. MDS disease severity should be considered, as severe disease with splenomegaly and splenic dysfunction may further contribute to infection risk independent of ruxolitinib [24, 25].

Opportunistic infection in patients with VEXAS syndrome can be attributed to neutropenia and neutrophil dysfunction, as well as those with associated MDS [2]. Bacteria are the most common cause of infection in patients with MDS treated with azacitidine [26]. Moreover, comorbidities with advanced age in MDS patients likely add to their susceptibility to infection [19, 27]. Interestingly, in a recent study evaluating 74 patients with VEXAS, MDS was not found to be an independent predictor of serious infection; instead, notable risk factors were as follows: advanced age at diagnosis (> 75 years), p.Met41Val mutation, arthralgia and treatment with JAKi [6]. Importantly, the p.Met41Val mutation is associated with a poorer prognosis, with higher severity cytopenias requiring regular transfusions [28, 29].

Recently, the burden of infections in VEXAS syndrome has been highlighted by EULAR, with recommendations for prophylaxis against *Pneumocystis jirovecii* and *alphaherpesvirus*, although the role of antifungal prophylaxis remains undefined [30]. This is likely due to the comparative rarity of cases and heterogeneity of affected patients. This is further complicated by antifungal drug interactions and the insidious nature of the disease, making both diagnosis and management difficult [31]. In contrast, antifungal guidelines are well established in other conditions, including HIV-acquired immunodeficiency syndrome patients with CD4 cell count < 200 cells/mm^3^, haematological malignancies and stem cell transplant recipients [15, 18, 32]. In retrospect, monitoring the CD4 count may have been beneficial in guiding infection prophylaxis for this patient, however concurrent immunosuppression from chronic corticosteroid exposure and intrinsic immune dysregulation may also affect the CD4 count, hence why this was not performed for our patient [33]. Recently, serum anti-granulocyte-macrophage colony-stimulating factor (GM-CSF) antibodies have been recognised to predispose to cryptococcal infection and can have prognostic implications [34, 35]. Although these autoantibodies are more commonly associated with *C. gattii* pulmonary infection, cases of *C. neoformans* meningitis have also been described, suggesting their utility in future cases [34, 35]. At our centre, the routine practice is to only order anti-GM-CSF antibodies if there are insufficient factors to contribute to cryptococcosis; however, in our case, the patient was thought to have substantial contributors, including underlying VEXAS syndrome with concurrent immunosuppression.

While our case has exemplified a rare infective complication in VEXAS syndrome, there are a few limitations. Given the novelty of VEXAS syndrome, treatment options were limited by available evidence and clinical experience. Allogeneic haematopoietic stem cell transplantation is also an emerging treatment option, though infections have also been reported as a complication [36]. Although azacitidine and transplantation were raised as possible options much earlier in his disease course, these were not pursued due to risk versus benefit evaluation at the time, including functional status, infection risk, cost and limited experience at this centre. Previously, azacitidine showed promise with variable efficacy, but recently been demonstrated to be effective regardless of MDS status [37, 38]. The patient has since been initiated on low-dose azacitidine and is tolerating well with some improvement in cytopenia (Fig. 3).

## Conclusion

We present a rare case of *C. neoformans* meningitis in a patient with treatment-refractory VEXAS syndrome without concurrent MDS. Given the significant morbidity and mortality associated with opportunistic infections, particularly cryptococcus infection, this case raises several important clinical practice points. Firstly, a multidisciplinary approach between immunology, rheumatology, infectious diseases and haematology specialties is required for prophylaxis and/or treatment with antifungal regimens in patients with VEXAS syndrome. Secondly, addition of such therapy needs to be considered on balance with the risk-benefit profile, with close monitoring of drug-drug interactions, degree of cytopenia, patient comorbidities and cumulative corticosteroid exposure. Finally, a growing need for a best practice consensus guideline on preventing opportunistic infections in VEXAS syndrome is urgently required to prevent irreversible sequelae.

## Data Availability

No datasets were generated or analysed during the current study.

## Abbreviations

BD: Twice daily
*C. gattii*: *Cryptococcus gattii*
*C. neoformans*: *Cryptococcus neoformans*
CrAg: Cryptococcal antigen
CRP: C-reactive protein
CSF: Cerebrospinal fluid
DMARD: Disease modifying antirheumatic drug
DVT: Deep vein thrombosis
ECOG: Eastern Cooperative Oncology Group
HIV: Human immunodeficiency virus
IFI: Invasive fungal infections
i.v.: Intravenous
JAKi: Janus kinase inhibitors
L-AMB: Liposomal amphotericin
MCV: Mean corpuscular volume
MDS: Myelodysplastic syndrome
MRI: Magnetic resonance imaging
PAN: Polyarteritis nodosa
PET: Positron emission tomography
PLT: Platelet count
p.o.: Per oral
s.c.: Subcutaneous
TMP-SMX: Trimethoprim/sulfamethoxazole
VEXAS: Vacuoles, E1 enzyme, X-linked, autoinflammatory somatic
VZV: Varicella zoster virus
WCC: White cell count
